# Adenovirus‐Associated Opsoclonus After Allogeneic Hematopoietic Stem Cell Transplantation

**DOI:** 10.1002/jha2.70259

**Published:** 2026-03-09

**Authors:** Toru Miyajima, Setsuaki Hoshino, Shingo Nojima, Souichi Shiratori, Taishi Iwanami, Takanori Teshima

**Affiliations:** ^1^ Department of Hematology Hokkaido University Faculty of Medicine and Graduate School of Medicine Sapporo Japan; ^2^ Department of Neurology Hokkaido University Faculty of Medicine and Graduate School of Medicine Sapporo Japan

**Keywords:** adenovirus‐associated hemorrhagic cystitis, allogeneic hematopoietic stem cell transplantation, opsoclonus

1

Opsoclonus is a rare oculomotor disorder characterized by chaotic, multidirectional saccades, typically associated with paraneoplastic or parainfectious autoimmune mechanisms [1]. While neurological complications may occur after allogeneic hematopoietic stem cell transplantation (allo‐HSCT), isolated opsoclonus triggered by viral reactivation is exceptional [2]. We report a case of opsoclonus associated with adenovirus‐associated hemorrhagic cystitis (AdV‐HC) after allo‐HSCT.

A 54‐year‐old man with acute myeloid leukemia in his second complete remission (CR) underwent allo‐HSCT from an HLA‐C allele‐mismatched unrelated donor. The conditioning regimen included fludarabine, busulfan, and 4 Gy total body irradiation. Graft‐versus‐host disease (GVHD) prophylaxis consisted of rabbit anti‐thymocyte globulin, tacrolimus, and short‐term methotrexate. Neutrophil engraftment was achieved on Day 12. Grade III acute GVHD involving the skin and gut occurred on day 16 but resolved with prednisolone, ruxolitinib, and mesenchymal stem cells.

On Day 113, he developed macroscopic hematuria and impaired renal function. AdV‐HC was diagnosed based on positive polymerase chain reaction (PCR) results in blood and urine. Although his urinary symptoms improved with hydration and reduction of immunosuppression, he developed severe dizziness approximately 3 weeks after the onset of AdV‐HC.

Neurological examination revealed chaotic, multidirectional, jerky eye oscillations consistent with opsoclonus, exacerbated by attempted pursuit (Figure 1, Supporting Information). Myoclonus and ataxia were absent. Brain magnetic resonance imaging was unremarkable, ruling out the possibility of posterior reversible encephalopathy syndrome or ischemic events. Cerebrospinal fluid (CSF) analysis revealed normal cell count, protein, and glucose levels; however, the presence of oligoclonal bands and an elevated IgG index indicated IgG synthesis within the central nervous system. CSF PCR for viruses, including herpes simplex virus, human herpesvirus 6, and AdV, was negative. A comprehensive workup for alternative etiologies, including paraneoplastic antibody panel (including anti‐Hu and anti‐Ri) and whole‐body computed tomography, was unremarkable. Bone marrow examination confirmed sustained CR of AML. At the onset of opsoclonus, acute GVHD was in CR and was quiescent under tapering immunosuppression with prednisolone 7.5 mg, tacrolimus 0.4 mg, and ruxolitinib 20 mg daily, without any concurrent exacerbations or newly emerging systemic manifestations.

**FIGURE 1:**
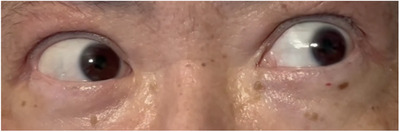
Multidirectional, high‐frequency, and involuntary conjugate ocular saccades (opsoclonus). See Supporting Information for video footage of this phenomenon.

Based on these findings, a diagnosis of parainfectious opsoclonus secondary to AdV infection was made. The patient received cidofovir for AdV‐HC, resulting in complete viral clearance. For the opsoclonus, he was treated with clonazepam, two courses of methylprednisolone pulse therapy, and intravenous immunoglobulin. His symptoms resolved almost completely approximately nine weeks after the onset of opsoclonus.

Opsoclonus is usually accompanied by ataxia and myoclonus as part of the opsoclonus–myoclonus syndrome (OMS), and is most often paraneoplastic in adults [1]. In this case, a paraneoplastic etiology was considered unlikely based on the negative results of comprehensive systemic evaluation and paraneoplastic antibody screening. Parainfectious OMS has been reported with various pathogens and is thought to arise from immune‐mediated mechanisms, in which molecular mimicry triggers an autoimmune attack on neuronal structures, leading to disinhibition of the oculomotor region of the cerebellar fastigial nucleus [3]. The delayed onset after AdV‐HC, evidence of intrathecal IgG synthesis, and favorable response to immunotherapy support an autoimmune mechanism rather than direct viral invasion.

To our knowledge, this is the first reported case of adult‐onset opsoclonus associated with adenovirus infection after allo‐HSCT. Early recognition of characteristic eye movements during recovery from acute viral infections, including AdV, is crucial, as timely immunotherapy can lead to substantial neurological recovery.

## Funding

The authors have nothing to report.

## Consent

Patient's consent was obtained for publication of the case.

## Conflicts of Interest

The authors declare no conflicts of interest.

## Supporting information




**Supporting File 1**: jha270259†sup†0001†Video.mp4

## Data Availability

This article contains all the data that was obtained during the research.
